# The effect of learning strategies on offloading decisions in response recall

**DOI:** 10.3758/s13421-025-01750-9

**Published:** 2025-07-11

**Authors:** Jenna R. Donet, Philip H. Marshall, Michael J. Serra

**Affiliations:** 1 Department of Psychological Sciences, Texas Tech University, 2700 18Th St, Lubbock, TX 79410 USA; 2 Department of Medical Education, Texas Tech University Health Sciences Center, Lubbock, TX USA

**Keywords:** Offloading, Learning strategies, Paired-associates

## Abstract

The decision-making processes involved in relying on an external source (cognitive offloading) for memory retrieval tasks have been discussed in numerous publications. The nature of original learning strategies could be an important contributing factor to the decision to offload but is unexamined. In this study we used a paired-associate learning task to investigate the influences of mnemonic (associative) and rote learning strategies on the likelihood of opting out, either by offloading or omitting a response. Further, we investigated the ways that certain outcome variables (specifically, the number of opt-out responses and amount of time spent studying) may in fact influence the relationship between learning strategy and decisions to persist in effortful search. We also investigated the degree to which any effects of learning strategy are specific to either offloading or omission decisions. Overall, we found a mnemonic learning effect of decreased frequency of general opt-out decisions relative to the rote group. Further, we found that mnemonic learning led to longer internal search times prior to opt-out responses, suggesting additional, intentional search processes such as trying to retrieve the original mnemonic, to help recover the response word. A partial mediation of the learning strategy effect on omission latency by performance factors suggested the learning strategy effect affects omission latency independently. Finally, relative to the rote learning strategy, the mnemonic strategy led to fewer instances of offloading, and longer decision latencies for omission responses.

## Introduction

Forgetting in the form of retrieval failure is a common occurrence in daily life. To account for such failure, external retrieval strategies, such as using Google Translate to recall a particular phrase in another language, might be a more attractive alternative to engaging in challenging internal memory searches. Numerous factors contribute to the decision to rely on an external store of information, a process known as *cognitive offloading* (Risko & Gilbert, 2016). Kelly and Risko (2022) recently showed that participants opted to utilize offloading as an encoding strategy when they knew they would have access to an external store for retrieval but opted to effortfully allocate study time to encode information when they anticipated no access to an external store at retrieval. Since participants used these learning strategies to lead to recall without offloading, we wanted to examine if the use of such learning strategies may be among the factors affecting the decision to offload during retrieval.

Cognitive offloading is an increasingly relevant topic of study, especially considering the growing access to technological advancements such as the Internet. Individuals can offload for any cognitive task (e.g., prospective memory or attention) and can also offload to any kind of externally based tool (e.g., finger counting or writing down to-be-remembered information) (Risko & Gilbert, 2016). With such variety in the application of offloading, the phenomenon has been investigated from several different perspectives, including different tasks, tools, and environments (for a review, see Risko & Gilbert, 2016). Offloading research thus far has major implications for the ways in which individuals offload using the Internet. Marsh and Rajaram (2019) suggested that the Internet is defined by characteristics including the display of fairly accurate information, consistently easy to access, and high user confidence in personal capability to search. Indeed, the Internet’s accessibility, reliability, and usability all increase the likelihood of its use as an optimal offloading tool. These conclusions about the Internet have been demonstrated in work on offloading, such as Storm and Stone’s (2017) finding that people are willing to forego the most efficient option in favor of offloading for information search tasks, and Ferguson and colleagues’ (2015) conclusion that access to the Internet leads to decreased willingness to provide responses in internal searches.

Of particular interest in the general offloading literature are the dynamics between internal and external stores for different types of memory. This investigative trend of offloading of encoding was sparked largely by Sparrow and colleague’s (2011) study pioneering the “Google effect,” which found that individuals who used an external store during encoding and expected to have access to it during retrieval showed drastically diminished performance when later only being able to retrieve from an internal store. Since then, this work has expanded to encompass two main lines of research: decisions to utilize offloading strategies for memory and the nuanced effects of utilizing offloading strategies on memory performance.

Large portions of the offloading literature have demonstrated that individuals typically opt to encode information to external storage when given the opportunity – even when it is nonoptimal, inconvenient, and when ceiling performance is possible without offloading (Ferguson et al., 2015; Gilbert et al., 2020; Risko & Dunn., 2015; Storm et al., 2017). However, recent work has suggested a more nuanced perspective of offloading behavior, indicating that participants consider the efficacy of internal and external memory stores for the task at hand, preferring, for example, internal storage of episodic memory and external storage for semantic memory (Finley & Naaz, 2023). Findings such as these have brought into focus the relevance of strategic decision-making work in studying cognitive offloading, which considers the costs and benefits of utilizing any one cognitive strategy. These perspectives consider inherently costly the effort expenditure necessary to engage in cognitive control and hold that perceived cost typically increases as time on task increases (Gray et al., 2006; Kool et al., 2010; Shenhav et al., 2017). For the most part, offloading studies have shown internal storage of information to be more effortful, suggesting that participants will opt for less effortful encoding unless motivated (by external rewards or intrinsic belief factors) otherwise (Gilbert et al., 2020; Sachdeva et al., 2020). As such, a current open question in the offloading field is what prompts individuals to select and persist in effortful memory strategies when less costly options are available.

When understanding the effects of offloading, it is important to consider what participants expect with regard to later access to an external store. Offloading is an effective strategy when current and later access to the sought-after store is reliable, consistent, and understandable (Storm et al., 2017; Weis & Weise, 2019, 2022; for discussion on effectiveness, see Kelly & Risko, 2022). When participants unexpectedly do not have access to the external store, most studies have shown varying levels of performance deficits in memory tasks (Eskritt & Ma, 2014; Gilbert, 2015; Hu et al., 2019; Kelly & Risko, 2019, 2022). Only recently have studies emerged looking into the nature of that deficit. For example, to investigate the reasons that memory performance may be poor for items offloaded during encoding, Kelly and Risko (2022) measured the effort that participants allocated to studying when they expected access to an external store and when they did not. They discovered that participants spend significantly less time studying word lists that they expect to have access to via an external store at retrieval. The authors suggested that participants used offloading as its own encoding strategy, and effortful learning for offloaded items did not take place, resulting in performance deficits for recall from internal-storage-only. In addition to these quantitative measures, the authors reviewed self-reported strategies used to learn word lists, finding that participants tended to spontaneously use encoding strategies when they knew they would not have access to external stores at retrieval. Though a variety of encoding strategies were reported by participants, the authors did not identify any differences in frequency of particular strategy use or in offloading behavior based on the usage of strategies. We felt that these questions were worth follow-up exploration and sought to pursue them in additional research.

Various learning strategies are more or less effective at different stages of learning (Hattie & Donoghue, 2016). Hattie and Donoghue (2016) divide learning into two active categories: the acquisition of knowledge and the consolidation of knowledge. During the acquisition phase, mnemonics can be particularly effective for students to grasp initial material in an organized and highly connected way (Bower, 1970, 1972; Roediger, 1980). Thus, at retrieval, high connectivity provides a greater likelihood the target memory may be retrieved. On the other hand, it appears that the application of rote rehearsal is effective particularly within the consolidation phase, where people practice that which they have already learned (Hattie & Donoghue, 2016), though it is sometimes used for encoding. The use of superficial maintenance rehearsal at encoding inevitably leads to shallow connectivity of the content, which can be ineffective at retrieval stages (Craik & Tulving, 1975; Hyde & Jenkins, 1973). Given this theoretical background, we wanted to know if encoding strategies that promote higher connectivity would lead to greater willingness to provide an answer and persist in internal searching behavior, despite the presence of offloading capabilities likely diminishing the threshold for both.

### The present study

To explore the effect of learning strategies on internal search persistence, we partially replicated Ferguson and colleagues’ (2015) methodology, which they used to measure for omission frequency and latency. In their work, the authors found that participants who had access to the Internet gave up on internal memory searches far more frequently and sometimes more quickly than those without Internet access. However, they did not have participants demonstrate the degree of prior knowledge before engaging in the experiment and therefore were not able to distinguish between learned but forgotten answers and entirely novel information. For example, if a presented question was entirely novel to a participant, they should recognize this unfamiliarity and decide to extend their search to the Internet much sooner. On the other hand, if participants encounter information for which they have already demonstrated learning, but experience immediate retrieval failure, they may search internally for more time before opting out. If some items, but not all, have already been learned, there may be an additional factor confounding the extension of searches to the Internet.

In order to account for this potential confound, we adjusted Ferguson and colleagues’ (2015) methodology to present entirely novel stimuli to participants in the form of manipulated linguistic word pairs and controlled for encoding variability by implementing encoding strategies and criterion learning, the latter two being novel procedures in the offloading literature. Thus, our general question of interest is whether different types of encoding strategies during learning influence offloading behavior when learning to a 100% criterion. To measure internal search behavior, we adopted Ferguson et al.’s (2015) dependent measures of frequency of opting out of answering and average time to opt out of an answer. Specifically, we manipulated encoding strategies by instructing participants to use either mnemonic associations or rote memorization to learn a list of linguistic paired associates.

In particular, mnemonic associations should be richly distributed throughout idiosyncratic semantic networks and allow a greater opportunity for highly connected encoding than the rote rehearsal (e.g., Adams et al., 1971; Gupta, 1985). Additionally, a robust effect has been found in many learning studies, showing that mnemonic learning is associated with better recall than rote learning (Adams et al., 1971; Atkinson, 1975; Pressley et al., 1982a). With effortfully encoded mnemonic associations, participants who cannot immediately recall the appropriate term may experience partial recall via the large number of mnemonic connections and persist longer in their effortful, internal search before electing to opt out. On the other hand, the rote group, when experiencing uncertainty in recall, may opt out more often, and sooner into the search. Hence, our two research hypotheses are:

- *H1:* Frequency of opting out will be comparatively higher for the rote group than for the mnemonic group.
- *H2*: The mnemonic learning group will search internally longer, yielding an overall longer latency before opting out than the rote learning group.

## Experiment 1

### Method

#### Participants

An a priori power analysis calculated through G*Power suggested 120 participants would be sufficient to identify relevant effects at a power level of 0.9, to a medium effect size (e.g., η_p_^2^ = 0.06, *d* = 0.5) (Faul et al., 2007; Furgeson et al., 2015). The present experimental design required participants to have a very good knowledge of the word pair list, resulting in many participants demonstrating perfect recall with no errors, only errors of one type, or lists unlearned within the allotted time. For example, a participant with no opt-out responses and no associated opt-out times could not have appropriate data for analysis. Therefore, all included datasets had at least one observation of correct responses, omission responses, and commission responses.

Our sample was 147 participants between the ages of 18 and 23 years, collected from the Texas Tech University subject pool. Participants were compensated with course credit. As the study trials were self-paced, we used a 90-min time limit and an eight study-test trial limit to ensure that disengaged participants did not end up in an endless loop of studying. Pilot data (*n* = 50) indicated that these limitations were sufficient for most participants to complete the study. Of the initial 147, 14 did not complete the study within the allotted 90 min (five in mnemonic, nine in rote), eight did not learn all word pairs within the eight-trial limit (five in mnemonic, three in rote), and one participant in the mnemonic group spontaneously switched to a rote learning strategy and was removed. One participant was removed due to a data collection malfunction. Therefore, an initial working sample of 123 participants was selected (34 men, one nonbinary). This study was reviewed and approved by the Texas Tech University Institutional Review Board (IRB).

#### Design

The primary manipulation applied to this study was a single-factor between-subjects design. Participants were randomly placed in either a mnemonic or a rote learning group. Response types were later examined as additional repeated-measures factors, resulting in mixed-subject analyses, to be presented in the *Results* section.

#### Materials

All word pairs are presented in Appendix A and full materials are available upon request. They consisted of a total of 24 English- and Dutch-derived word pairs. We elected to use linguistic word pairs to mimic an easily generalizable recall task like language learning. The use of a Google Translate facsimile presented a reliable opportunity as our offloading resource.

**Fig. 1:**
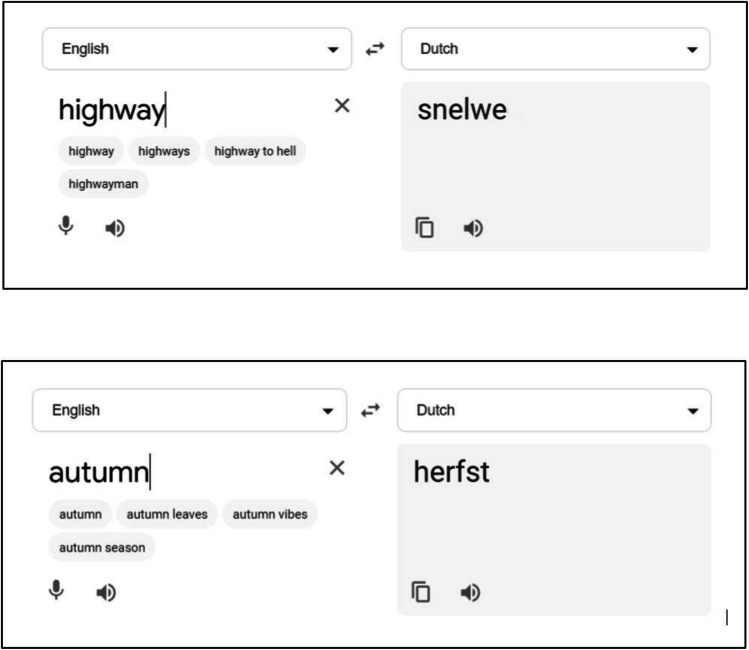
Examples of the Google screenshots provided after an omission response on the final test. The Dutch association of “highway” has been truncated to six letters and the presented screenshot edited. By comparison, the second provided screenshot of the Dutch association of “autumn,” which only had six letters to begin with, was therefore unedited

Words pairs were selected from a Dutch dictionary of common words (De Pau, 2014). We truncated all Dutch words to six letters in length to more readily promote learning, then edited screenshots of the Google Translate page for the word pair using Microsoft Paint to reflect these changes (see Fig. 1). English cue words ranged in length from three to 12 letters. Each participant was required to learn a list of 20 word pairs randomly drawn for that participant from the available list of 24 word pairs. When presented with the English word, participants were required to respond with the correct pseudo-Dutch word.

Both experiments were presented in the laboratory testing room on Intel Core i7 computers with Acer 27-in. displays, presenting material using a Qualtrics program.

#### Procedure

Participants were first instructed in the kind of learning strategy they were to use. The rote learning group was instructed:

“To learn by rote, retype each provided pair as many times as you need to feel confident that you know the pair. This would be like saying it over and over to yourself. Retype and repeat each word pair until you are confident you will be able to recall the pair later.”

Alternatively, the mnemonic group was instructed:

“To learn by association, try to come up with something meaningful that links the two words. It could be through sound, imagery, concept, or anything you can come up with. It doesn’t matter how silly the association may seem to be as long as it is meaningful to you and you are confident it will help you remember the word pair. For example, for the word pair chicken-kip, I could say that baby chicks say ‘cheep!’ which sounds like kip (pronounced as ‘keep’). I could also say ‘chicken coop’.”

**Fig. 2:**
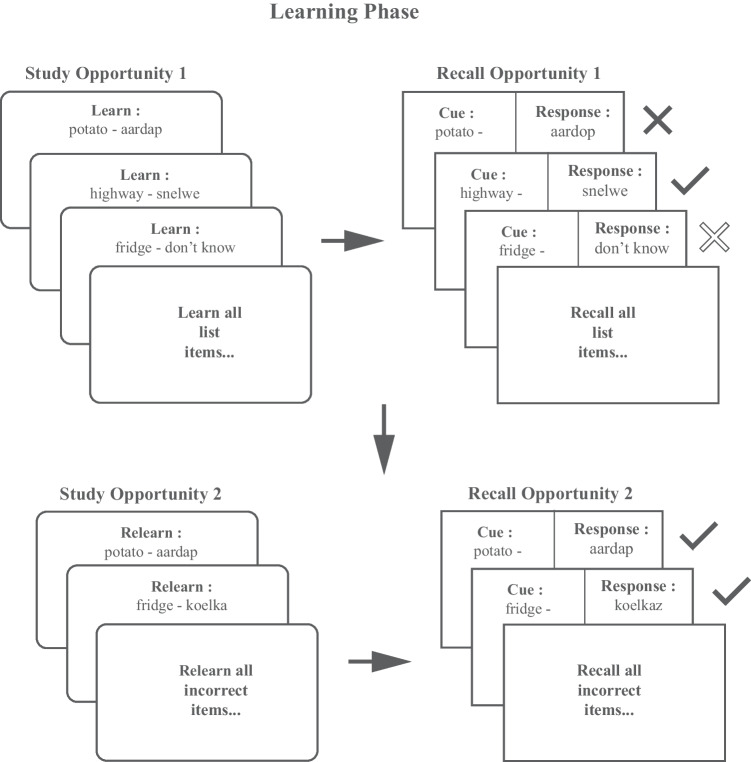
Schematic of the procedure during the learning phase. The figure represents the drop-out learning procedure. Participants began by learning a list of items, attempting to retrieve them, then restudying and retrieving only those items that were initially incorrect. This cycle was repeated, with each following iteration only containing those incorrect items from the previous iteration until the participant correctly retrieved all items once. The feedback indicated in this schematic is for readers’ benefit and was not provided to participants

After these corresponding instructions, both groups then engaged in eight possible learning trials. Each learning trial had two self-paced components, a study opportunity, and a recall opportunity, as depicted in Fig. 2. During the study opportunity, participants were randomly presented with the word pair and, depending on their assigned learning strategy, a space to type either their mnemonic or repetitions of the word pair. After clicking the arrow to proceed, participants then provided a judgment of learning (JOL) on a 1–5 Likert scale to indicate their likelihood of recall on a later test.^1^ This was a sliding scale with the slider anchored at one, labeled “Not at all confident,” while five was labeled “Very confident.” Once they proceeded, they were presented with the next word pair, and the cycle was repeated until all 20 word pairs had been studied. For each associated recall opportunity during the learning phase, participants were randomly presented with a cue word they had studied and asked to retrieve the answer. Participants were prompted to either provide the answer or opt-out of an answer by clicking “Don’t know.” After attempting to recall each Dutch word, participants started the next learning trial, wherein only any word pair with an error of omission or commission on the previous recall trial was restudied in a random order. Thus, for the learning phase of the study, participants engaged in a randomly ordered drop-out learning task, ensuring that participants had one correct response for each pair, and had thus learned the list to a criterion of 100%. Participants were not provided with direct feedback during the learning stage; their only indication that they were incorrect on their answers was the eventual restudy of that pair.

After demonstrating learning for the entire list, participants engaged in a 5-min retention interval task. For this task, participants were required to solve arithmetic problems presented without feedback. These problems were not scored, but participants’ activity was monitored by experimenter observation to ensure continued engagement with this task.

For the final testing phase, participants engaged in a procedure very similar to the recall opportunity during the learning phase. They were informed:

“For this final recall test, we are going to present you with each English word and you will be asked to provide the target word. Please try your best to recall the Dutch word, but if you cannot remember, you may click ‘Don’t know’ to access a portion of a Google page that will provide you with the correct answer, like the example below. You will then type the correct answer into the provided text box.”

**Fig. 3:**
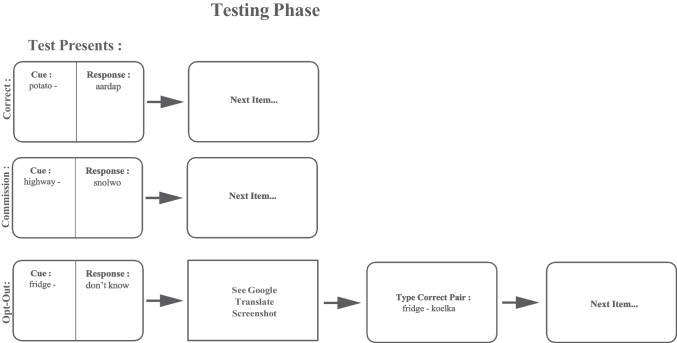
Schematic of the procedure during the final testing phase. The figure represents the three outcome options for the final testing phase of the procedure. When prompted to retrieve the response word, correct responses and errors of commission both triggered immediately moving on to the next item. For any response of omission (“Don’t know”), participants received access to the Google screenshot, provided the response word, and then proceeded to the following item. Any feedback indicated in this schematic is for the reader’s benefit and was not provided to participants

Each cue word was presented individually in a randomized order, prompting participants to either respond with the response word in the text box or click “Don’t know.” Similar to Ferguson et al. (2015), an omission response immediately presented participants with an opportunity to offload. However, instead of actively searching the Internet for the answer, an edited screenshot of a simulated Google Translate page for that word pair only (see Fig. 3) was provided. We elected to use edited screenshots and omit the actual searching process to maintain the consistency of the offloading conditions for all participants. To maintain veracity of the task, after offloading, participants were also prompted to provide the response word as given in the screenshot. Correct responses and errors of commission did not force users to offload and did not provide any sort of feedback to participants.

After completing the final testing phase, participants answered a short demographic questionnaire on age, gender, academic status, and Dutch and German experience, were thanked, and dismissed.

### Results

Main dependent variables of interest were participants’ final test response times averaged by response type (e.g., correct, opt-out, commission), final test performance by summed responses of each type (correct, opt-out, commission), and the summed time participants spent studying, yielding a total of seven dimensions.

#### Data treatment

The initial 123 participants were screened for outliers on all seven dependent variables using the boxplot method, which is based on Tukey’s interquartile range (IQR) approach (Tukey, 1977). By this approach, a datapoint was considered an extreme outlier if it exceeded three times beyond the first and third quartiles (IBM Corp., 2023). This procedure indicated 13 extreme univariate outliers, which were removed from the dataset (one on number of opt-outs, two on correct latency, two on opt-outs latency, and eight on commission latency). Following this initial removal of outliers, we assessed for normality. Five variables (opt-out count, commission count, correct latency, opt-out latency, and commission latency) were significantly negatively skewed (> 3.2 standardized; Kim, 2013) and those used in analyses requiring normality were corrected using logarithmic transformations (opt-out count, correct latency, opt-out latency, commission latency). The count of correct responses demonstrated a significant positive skew but was analyzed through chi-squared test of independence and was not transformed. We then removed all moderate outliers that appeared on more than one variable, identified as any data point that fell more than 1.5 times the IQR below the first quartile or above the third quartile (six cases), and screened for multivariate outliers, removing two cases that exceeded the threshold value of 20.52 on Mahalanobis distances. Following these procedures, data were approximately normal and displayed minimal influence due to outliers, with an acceptable observed power level of 0.81 on our most conservative analyses. The data of nine participants who reported previous experience with Dutch or German words were retained for analysis, since by simple observation no performance or behavioral anomalies were observed. All these changes resulted in a dataset of 102 participants, 49 in the mnemonic group and 53 in the rote group.^2^

For within-subjects measures, Greenhouse–Geisser corrections were applied to correct for violations of sphericity. The learning strategy categorical variable was coded with the mnemonic group as 0 and rote as 1.

#### Statistical analyses

The mnemonic group (*M* = 42.26 min, *SD* = 11.81 min) spent significantly longer completing the study than the rote group (*M* = 35.45 min, *SD* = 10.85 min), *t*(100) = 3.04, *p* < 0.003, *d* = 0.60 Tables 1, 2 and 3.

**Table 1:** Count, expected count, and adjusted residuals for performance chi square

Learning strategy		Correct	Opt-outs	Commission
Mnemonic	Count	723	109	148
	Expected count	696.1	146.0	137.9
	Adjusted residual	2.6	−4.6	1.3
Rote	Count	726	195	139
	Expected count	752.9	158.0	149.1
	Adjusted residual	−2.6	4.6	−1.3

**Fig. 4:**
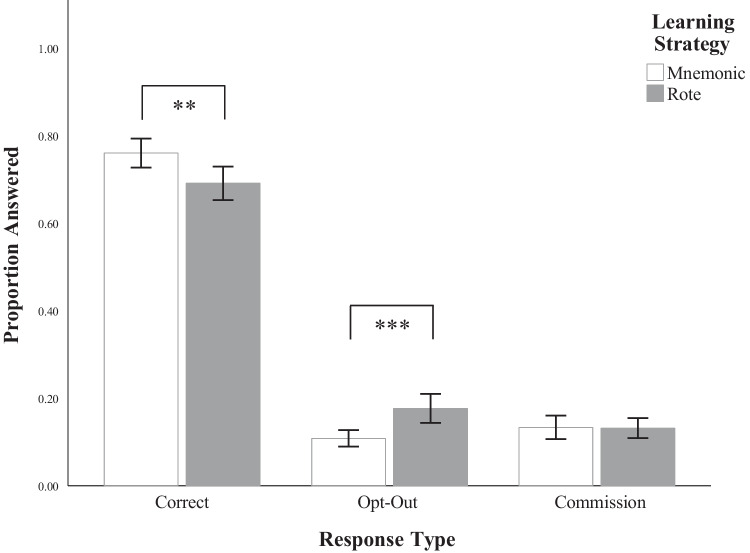
Mean proportion of responses by response type and learning strategy. The figure depicts the proportion of each question answered to total available questions. Error bars represent ± 1 *SE*. **.** = 0.05 > *p* < 0.10; * = *p* < 0.05; ** = *p* < 0.01; *** = *p* < 0.001

For every participant, each observation of correct, opt-outs, and commission responses was considered an independent trial, resulting in the count presented above. For average and proportional performance, refer to Table 2 and Fig. 4, respectively. Standardized residuals resulted in a similar outcome, but violation of independence was driven primarily by group differences in opt-out responses only

**Table 2:** Mean number of word pairs by learning strategy and response type

Average Performance			
Group	Correct	Opt-out	Commission
Mnemonic	14.76 (0.40)	2.22 (0.20)	3.02 (0.31)
Rote	13.70 (0.41)	3.68 (0.37)	2.62 (0.23)
Total	14.21 (0.29)	2.98 (0.23)	2.81 (0.19)

Values are given as means, with standard errors in parentheses

**Table 3:** Response time by learning strategy and response type in standard seconds

Average Response time				
Group	Correct	Opt-out	Commission	Total
Mnemonic	1.67 (0.14)	13.75 (1.29)	1.75 (0.19)	5.72 (0.54)
Rote	1.64 (0.12)	8.41 (0.71)	1.69 (0.23)	3.91 (0.35)
Total	1.66 (0.09)	10.97 (0.77)	1.72 (0.15)	-

Values are given as means, with standard errors in parentheses

#### Opt-out frequency

In order to identify if the frequency of opting out was related to learning strategy, we ran a Pearson chi-squared test of independence measuring final test performance through response frequency as a function of learning strategy and response type (correct, opt-outs, commission error). This analysis showed a significant relationship between learning strategy and response type, *χ*^*2*^(2) = 21.51, *p* < 0.001. A post hoc test of adjusted standardized residuals, depicted in Table 1, revealed that violation of independence between the two variables was largely driven by group differences in opt-out responses (*p* < 0.001) and correct responses (*p* = 0.008), such that the rote group had more opt-out responses and fewer correct responses than the mnemonic group. Frequency of responses of commission did not significantly vary between groups. The odds ratio of responding with an opt-out rather than a correct or commission response when using a rote learning strategy is 1.46, 95% confidence intervals (CI) [1.12, 1.9] times higher than those using a mnemonic learning strategy. For more specific descriptives regarding performance, refer to Table 2, and for the proportion of each response type, refer to Fig. 4. These results support *H1*.

#### Opt-out latency

**Fig. 5:**
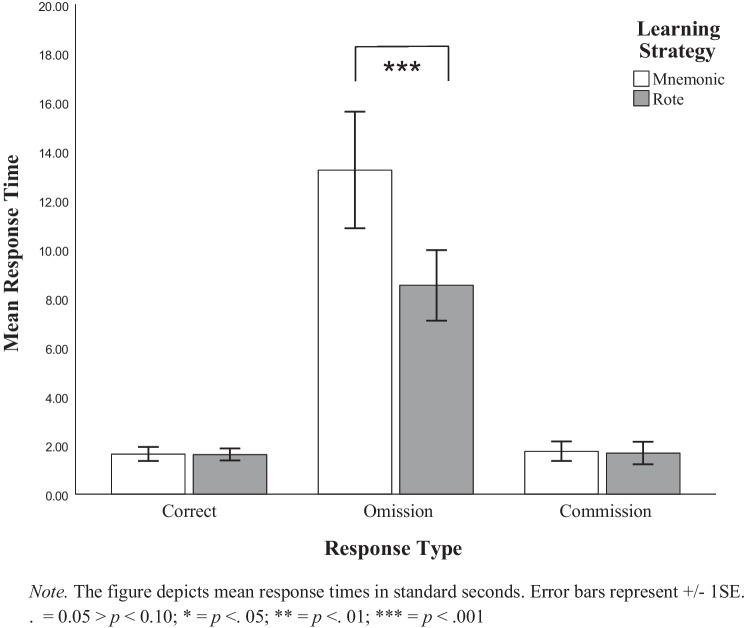
Mean response time by response type and learning strategy. The figure depicts mean response times in standard seconds. Error bars represent ± 1 SE

To establish the influence of learning strategy and response type on final recall response time, a 2 × 3 mixed-model ANOVA was used to determine the between-subjects effects of mnemonic and rote learning on log-transformed within-subjects effects of correct, opt-out, and commission response latencies. Descriptive data from this analysis are presented in Table 3 and Fig. 5*.* The main effect of learning strategy was significant, such that the mnemonic group searched longer before responding than the rote group did, *F*(1, 100) = 8.15,* p* = 0.005, η_p_^2^ = 0.08. There was also a significant main effect of response latency type, such that opt-out latencies were significantly longer than correct and commission latencies, *F*(1.68, 167.58) = 361.11, *p* < 0.001, η_p_^2^ = 0.78. Pairwise comparisons indicated that opt-out decisions took, on average, 9.32 more seconds than correct responses, *t*(99) = 2.25, *p* < 0.001, *d* = 3.22, and 9.26 more seconds than responses of commission errors, *t*(99) = 2.02, *p* < 0.001, *d* = 2.84. No significant differences between correct response times and commission error response times were identified. Lastly, learning strategy and response type interacted to influence learning strategy differences by response type, *F*(1.68, 167.58) = 7.08, *p* = 0.002, η_p_^2^ = 0.07. Pairwise comparisons indicated 5.35-s learning strategy differences in response latencies for opt-out responses, *t*(99) = 3.91, *p* < 0.001, *d* = 0.39, but no significant learning strategy differences for correct or commission error response latencies, *p*s > 0.05.

These results are consistent with the expectation that participants conducted an internal search prior to eventually opting out and that they searched comparatively longer in the mnemonic group than the rote group before extending the search to Google Translate, presumably taking time to retrieve and decode the mnemonic, lending support towards *H2*.

. = 0.05 > *p* < 0.10; * = *p* < 0.05; ** = *p* < 0.01; *** = *p* < 0.001.

Research has suggested that the decision to offload is partially informed by online task performance, including accuracy and time spent on task (Dunn & Risko, 2019; Dunn et al., 2019; Gray et al., 2006; Weis & Kunde, 2024). These papers indicate that as participants are aware of poor performance, they are more willing to offload rather than expend additional effort and time persisting in fruitless searches. To ensure that the learning strategy effect on response time is not primarily due to other aspects of performance, we ran an initial t-test to identify group differences in study time, and then examined a follow-up mediation model, to represent the degree to which the learning strategy effect on average opt-out latency is due to the group differences in frequency of opt-outs or overall study time. A full mediation would suggest that the time participants spent internally searching prior to search extension was shorter because overall task performance was considered low due to incorrect responses and overall time spent on task. A partial mediation or no mediating effect of opt-out frequency would suggest that the learning strategy used independently affects internal search time prior to opting out. The t-test indicated significant group differences in study time, *t*(100) = −3.31, *p* < 0.001, *d* = −0.66. with the rote group (*M* = 28.07 min, *SD* = 10.85 min) studying for longer than the mnemonic group (*M* = 21.07 min, *SD* = 8.14 min). In tandem with the overall longer time for the mnemonic group in the study, we find that the mnemonic group took longer on the final test than the rote group, ultimately accounting for the differences between total time on task and study time.

**Fig. 6:**
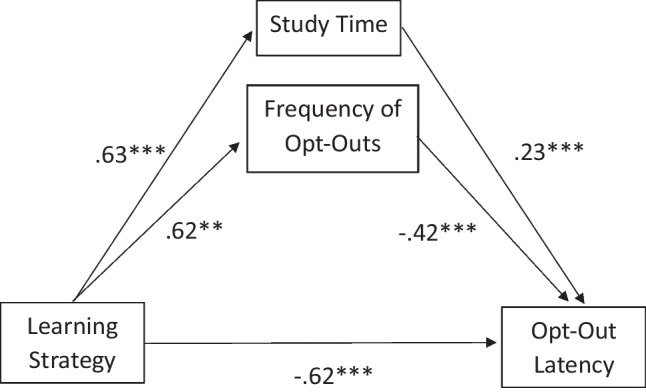
Representation of pathways of number of opt-outs and overall study time mediating the relationship between learning strategy and opt-out latency. The figure reports standardized beta weights. Learning strategy was coded with the mnemonic group as the reference group. **.** = 0.05 > *p* < 0.10; * = *p* < 0.05; ** = *p* < 0.01; *** = *p* < 0.001

This mediation model was examined using the PROCESS Macro (Hayes, 2013), with 5,000 bootstrap samples and 95% CIs, and is represented in Fig. 6. The mediation model predicted opt-out latency over and above the total effect model, *F*(3, 98) = 15.13, *p* < 0.001, accounting for 32% of the total variance in opt-out latency. Being in the rote group led to longer study times,*a*_1_ = 376.39 (seconds), *t*(100) = 3.31, *p* = 0.001, 95% CI [150.88, 601.91]. According to unstandardized *b*-weights, the rote group spent 7 more minutes to reach criterion than did the mnemonic group. In turn, timeC longer search times, *b*_1_ = 0.0002, *t*(100) = 2.64, *p* = 0.010, 95% CI [0.0001, 0.0004]. In other words, for each 10 min spent studying, participants tended to spend 1.73 s on average persisting before opting out. Being in the rote learning group significantly predicted more opt-outs,* a*_2_= 0.30 (logged frequency), *t*(100) = 3.27, *p* = 0.002, 95% CI = [0.18, 0.48]. Specifically, being in the rote group resulted in an average of 1.39 more instances of opting out than the mnemonic group. Number of opt-outs significantly predicted opt-opt out latency, *b*_2_ = −0.50, *t*(100) = −4.70, *p* < 0.001, 95% CI [−0.71, −0.29]. For every additional instance of opt-out responses, the average internal search time decreased by 1.58 s. Lastly, there was still a direct effect of learning strategy, *c’* = −0.36, *t*(100) = −3.39, *p* = 0.001, 95% CI [−0.57, −0.15], such that being in the rote group independently decreased the average internal search time by 1.65 s relative to the mnemonic group. However, both study time (*ab*_1_ = 0.09, *SE* = 0.04, 95% CI [0.01, 0.17]) and number of opt outs (*ab*_2_ = −0.15, *SE* = 0.05, 95% CI [−0.25, −0.06]) had significant indirect effects on opt-out latency, and reduced the total effect of learning strategy. Thus, results show an indirect effect of learning strategy on opt-out latency, by affecting the frequency of opt-out responses and overall study time. However, even after accounting for the group effects on performance variables, learning strategy still exhibited a significant direct effect on opt-out latency, suggesting a partial mediation effect (Baron & Kenny, 1986). Taken together, these results suggest that while the mnemonic learning group searched longer prior to opting out with a corresponding performance outcome, simply encoding items with a mnemonic learning strategy led to individuals persisting longer prior to opting out.

## Discussion

Experiment 1 investigated the effect of utilizing different learning strategies on the frequency of opt-out responses and time to decide to opt out in the presence of an offloading paradigm. Addressing *H1*, in accordance with previous literature (Adams et al., 1971; Atkinson, 1975; Atkinson & Raugh, 1975; Gupta, 1985; Pressley et al., 1982b), we identified a learning effect of better performance for recall of linguistic word pairs with a mnemonic rather than rote learning strategy. In this experiment, the learning strategy effect on performance was accompanied by an interaction that suggested greater numbers of correct responses and fewer opt-out responses in the mnemonic group than the rote group, with no difference in errors of commission. In other words, the learning strategy seemed to affect the willingness to opt out from answering, rather than any other behavior regarding errors of commission.

Data supporting *H2* indicate that overall, participants took longer to opt out when in the mnemonic group relative to the rote group, presumably because they were searching for longer. However, when accounting for response type, learning strategy differences appeared to be due to persisting in an internal search before electing to opt out, diminishing the likelihood that this effect was due to different retrieval strategies or the increased time to recall the mnemonic followed by the target word. Additionally, we found that participants had longer response times for items they eventually opted out from than for items they answered, whether correctly or incorrectly. This result suggests that participants were likely engaged throughout the duration of the final test. Discussing the potential for performance effects on opt-out latency, the mediation model presented in Fig. 6 holds that the effect of learning strategy on opt-out latency is partially explained by the effect of learning strategy on performance and study time. In turn, performance and study time affect opt-out latency itself. Regardless, the role of performance in predicting offloading outcomes did not entirely mediate the learning strategy effect.

The present results and conclusions serve as a promising beginning in the exploration of the effects of learning strategies on persisting in internal search. However, without an option to opt out of the trial without offloading, participants are forced to offload, rather than strategically electing to do so. Thus, clarification is necessary to identify the effect of the learning strategy as particular to the offloading experience, rather than any experience of opting out. We therefore present a second experiment with the goal of investigating the offloading experience relative to those of true omissions.

## Experiment 2

### Method

#### Participants

We recruited 132 Texas Tech students (41 men, two nonbinary, age *M* = 19.42 years, *SD* = 1.25 years), to participate in this experiment from the same participant pool as in Experiment 1. Sample size was determined in order to replicate the effect size of Experiment 1, at a power level of 0.8. This study was approved by the Texas Tech IRB, and all participants provided informed consent. None had participated in Experiment 1.

#### Design, hypotheses, and materials

This study utilized a 2 × 3 × 2 mixed-subjects design. The between-subjects factor was two levels of learning strategy: mnemonic or rote learning, randomly assigned to participants. In order to investigate how the learning effect may be specific to offloading, we introduced two new within-subjects factors. First, we created three within-subjects final test blocks (offload only, omit only, and choice) in order to directly compare decisions of offloading and omission. Second, we added another within-subjects factor (studied, unstudied) to ensure that participants were offloading and omitting intentionally, by presenting six word pairs participants had never studied, in addition to the original 24 studied word pairs. We expected that participants would be able to quickly identify their unfamiliarity with these word pairs and opt out accordingly, while thinking longer for word pairs they were familiar with.

With the goal of replicating our original findings, we present the following hypothesis:

- *H3:* The offloading-only block should replicate the effects of Experiment 1, indicating a greater rate of offloading and shorter response times for the rote learning group.

To effectively dissociate the learning effect on opt-out decisions and strategic offloading decisions, results must support one or both of the following hypotheses:

- *H4a:* The omission-only block has fewer instances of opting out and significantly different response times relative to the offloading-only block and the choice block.
- *H4b:* The choice block should show a preference for offloading over omitting, a greater likelihood to omit unstudied word pairs than studied, and greater frequencies and shorter latencies of offloading in the rote group.

Materials were altered in order to increase the number of word pairs participants learned, but not to increase the overall difficulty of the study. Thus, participants now saw a total of 30 word pairs: 24 learned throughout the experiment, and six unstudied word pairs on the final test. Also, rather than six-letter target words, each target word in Experiment 2 consisted of four letters.

#### Procedure

**Fig. 7:**
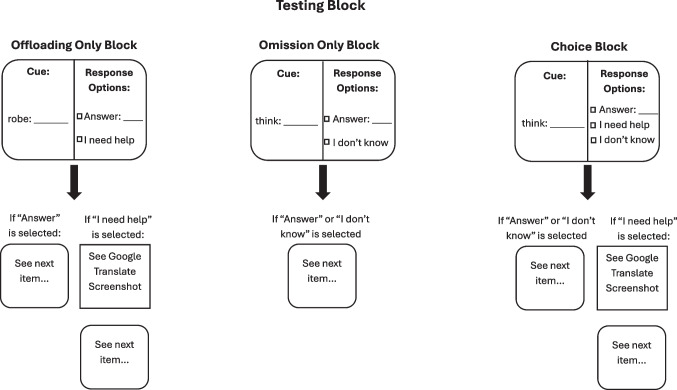
Schematic of the procedure during the final testing phase of Experiment 2. The figure describes the altered procedure for the final test of Experiment 2. Three blocks were presented, each with ten word pairs. Not depicted is that participants were required to provide an answer if they clicked “Answer,” and after viewing the Google Translate screenshot, as was the case in Experiment 1. Similarly, correct and commission responses were both possible and handled the same way as in Experiment 1

Overall, the learning procedure was very similar to that of Experiment 1, with three main exceptions. The first was that participants did not experience any confidence measures, focusing Experiment 2 on questions arising from Experiment 1. Secondly, participants only engaged in three learning and recall cycles, not being held to a 100% learning criterion. This change was to increase the overall corpus of error behavior that we could use in the analysis. The third major change was made to the final test. Rather than one large final test, we divided the list of word pairs into three lists of eight studied word pairs, reflecting the three response options, and two unstudied word pairs. These lists were randomly assigned to blocks for each participant. To review, the three final test blocks, presented in Fig. 7, varied depending on whether participants were able to select an offloading paradigm that presented an edited Google Translate screenshot. All blocks allowed participants to provide the target word if they knew it. The offload-only block presented the choice to either answer or offload through the “I need help” option. On this block, an “I need help” response would show the participant the Google Translate screenshot, which was defined as offloading. Participants were informed: “For this list, you will be able to access help, but you cannot skip words. If you feel you cannot recall the word, you may click ‘I need help’ to access a portion of a Google Translate page that will provide you with the correct answer.” The omission-only block presented the choice to answer or omit through the “I don’t know” option, which led to no screenshot. For this block, participants were informed: “For this list, you will not be able to access help, but you can skip words. If you feel you cannot recall the word, you may click ‘I don't know’ to skip that question.” The choice block presented the choice between all three: answer, offload, or omit, and informed participants: “For this list, you will be able to access help and skip words. If you feel you cannot recall the word, you may click either ‘I need help’ to access the Google Translate screenshot, or ‘I don't know’ to skip that question.” The choice block was always presented last, in order to give participants the opportunity to experience both offloading and omission blocks. Offload and omission block orders were randomly determined.

After providing informed consent, allowing them to withdraw from the study at any time without penalty, and providing demographic information, participants studied 24 word pairs throughout three learning and recall trials. These trials utilized the same drop-out learning paradigm as presented in Experiment 1. After either recalling all word pairs once or reaching the end of the three learning cycles, participants moved on to a 5-min retention interval task, where they were asked to solve arithmetic problems. For the final test, participants then either experienced the offloading block followed by the omission block, or vice versa. After completing both, participants experienced the choice block. Once participants finished the choice block, they were thanked and excused.

### Results

#### Data preparation

All data were prepared and analyzed using R version 4.4.2 (R Core, 2024), with main analyses conducted through the lme4 package (Bates et al., 2015). These models included three main categorical predictors: learning strategy, with mnemonic learning as the referent; final test block type, with the offloading block as the referent; and study status of word pairs, with studied word pairs as the referent. Trial outcomes were coded on two variables. First, to predict the likelihood of opt-out behavior (including both offloading *and* omitting) each trial was coded as 1 for opt-out responses and 0 for any correct or commission error responses. Second, to assess specific distribution of types of responses on each block, each trial was coded as a 0 for offloading, 1 for omission, 2 for correct, and 3 for commission. Response time was measured as a continuous dependent variable, measured from the start of the trial to the participants’ last click, and was analyzed only for trials in which participants opted out (min = 0.31 s, max = 77.85 s). Outliers were only assessed for response time and were first identified at a trial level utilizing Cook’s Distances (Bollen & Jackman, 1990). Out of 1,456 opt-out trials, 54 were identified as influential and removed from the analysis. Outliers at the participant level were identified as those with response times greater than three standard deviations from the mean; eight participants met this criterion and were excluded. This resulted in a total of 124 participants, for which a slight positive skew on response time was observed. However, linear mixed-effects models are generally robust in the presence of non-normality on dependent measures (Arnau et al., 2013), and all model assumptions were met.

#### Analyses

The mnemonic group (*M* = 56.76 min, *SD* = 18.61 min) took longer to perform the task than the rote group (*M* = 53.58 min, *SD* = 18.49 min), *t*(129) = 4.22, *p* < 0.001, *d* = 0.74. In addition, the mnemonic group (*M* = 70.51 s, *SD* = 29.96 s) took longer on each block in the final test than the rote group did, (*M* = 62.18 s, *SD* = 29.31 s), *t*(391) = 2.78,* p* = 0.006, *d* = 0.28. Data representative of the distribution of all final recall test responses are presented in Table 4.

**Table 4:** Average number of responses by response type, learning strategy, study status, and test block

Learning strategy	Study status	Final test block	Offload	Omission	Correct	Commission
Mnemonic	Studied	Offload	1.12 (1.61)	-	5.76 (2.16)	0.97 (1.35)
		Omit	-	0.83 (1.57)	5.85 (2.08)	1.22 (1.40)
		Choice	1.1 (1.47)	0.45 (0.77)	5.4 (2.09)	0.98 (1.14)
	Unstudied	Offload	1.75 (0.51)	-	-	0.17 (0.46)
		Omit	-	1.83 (1.57)	-	0.07 (0.31)
		Choice	1.25 (0.84)	0.65 (0.80)	-	0.07 (0.36)
Rote	Studied	Offload	2.15 (2.27)	-	4.52 (2.60)	1.28 (1.82)
		Omit	-	2.03 (2.11)	4.89 (2.31)	0.92 (1.23)
		Choice	1.92 (2.09)	0.74 (1.65)	4.58 (2.59)	1.7 (1.38)
	Unstudied	Offload	1.78 (0.57)	-	-	0.17 (0.55)
		Omit	-	1.89 (0.36)	-	0.08 (0.32)
		Choice	0.71 (1.22)	0.75 (0.88)	-	0.08 (0.37)

Table 4 depicts the average response count in each category. These averages were calculated by being summed at a participant level, then averaged at a group level. Omissions were not possible in the offload block, and offloads were not possible in the omission block. No correct responses were observed on the unstudied questions, as the stimuli were entirely novel. Values are given as means, with standard errors in parentheses

#### Offloading and omission frequency

**Fig. 8:**
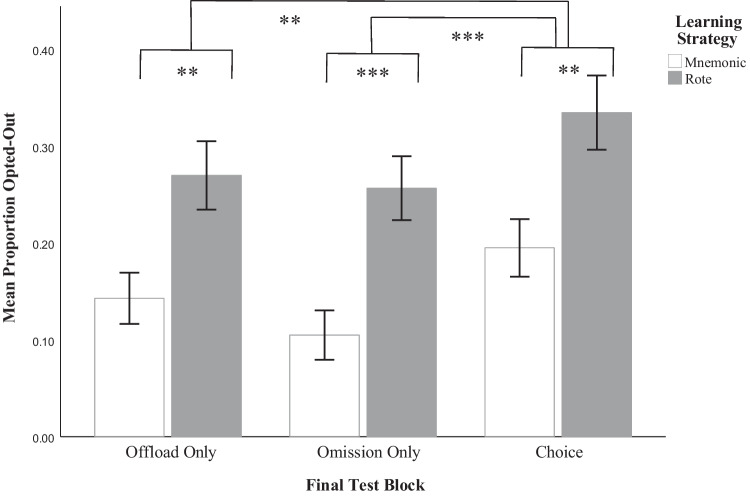
Proportion of opt-outs by block and learning strategy. The figure depicts the proportions of word pairs opted out by test block and learning strategy and is only representative of studied word pairs. Offloads and omissions have been combined in the choice block, to depict the results of the proportion analysis. For a full breakdown of the number of offloads and omissions in the choice block, refer to Table 4. Error bars represent ± 1 SE. **.** = 0.05 > *p* < 0.10; * = *p* < 0.05; ** = *p* < 0.01; *** = *p* < 0.001

In order to investigate the likelihood of opting out on studied word pairs as predicted by learning strategy and final test block, we calculated the proportion of opting out on each block by averaging the binary coding of opt-outs and analyzed these proportions through a linear mixed-effects ANOVA. Data from this analysis are presented in Fig. 8. We elected not to include unstudied word pairs in this analysis since participants almost always opted out on these and would therefore inflate the proportions for the variable of interest. A significant main effect of learning type showed that opt-out decisions were more frequent in the rote group than in the mnemonic group, *F*(1, 123) = 12.02, *p* = 0.001, η_p_^2^ = 0.09, with pairwise comparisons indicating that this effect occurred in the offloading block (*t*(192) = −2.90,* p* = 0.006, *d* = −0.20), the omission block (*t*(192) = −3.35, *p* = 0.001, *d* = −0.24), and the choice block (*t*(192) = −3.08, *p* = 0.002, *d* = −0.22). There was also a main effect of testing block on proportion of offloading, *F*(2,246) = 11.29, *p* < 0.001, η_p_^2^ = 0.08. Pairwise comparisons showed that opt-outs were more frequent in the choice block relative to the offloading block (*t*(192) = −3.22, *p* = 0.004, *d* = −0.21) and the omission block (*t*(192) = −4.64,* p* <. 001, *d* = −0.30), but that there was no difference between the offloading and omission blocks.

To specifically investigate the differences between omissions and offloading in the choice block, we only analyzed the trials from the third block that were coded as an offload or an omission. Those trials were entered into a 2 × 2 chi-squared test of independence that assessed the distribution of offloading and omission responses across studied versus unstudied word pairs, presented in Table 5. The chi-squared test demonstrated a significant deviation from expectation, χ^2^(1) = 4.57, *p* = 0.033. Post hoc standardized residuals demonstrated that each cell significantly deviated from the default expectation by 2.14 standard deviations, and participants more frequently responded with offloading than they did with omissions. However, the odds of offloading were 1.23 times greater when the word pair was studied than when it was unstudied, and the odds of omissions were 1.17 times higher when the word pair was unstudied than when it was studied. This suggests that participants effectively differentiated between offloading and omission opportunities and were more willing to omit a response for unstudied word pairs than they were for studied. Further, the odds of offloading in general were 1.5 times higher than omitting, suggesting that participants had a general preference for offloading in the choice block. Lastly, as represented in Table 6, we conducted a secondary 2 × 2 chi-squared test of independence to investigate the distribution of offloading and omissions in the choice block across learning strategy, which, with nonsignificant results (*p* > 0.05), suggested the frequency of offloading and omissions did not deviate from default expected values across learning strategies. Thus, the ratio of offloads to omissions was the same for each learning group, suggesting learning strategy did not result in one group omitting far more than the offloaded. Rather, omitting was equally unlikely in each learning group.

**Table 5:** Count, expected count, and adjusted residuals for opt-outs by study status

Learning strategy		Offloads	Omissions
Studied	Count	191	75
	Expected count	179.8	86.2
	Adjusted residuals	2.1	−2.1
Unstudied	Count	149	88
	Expected count	160.2	76.8
	Adjusted residuals	−2.1	2.1

Table 5 depicts the values and outcomes of the chi-squared test of independence assessing the distribution of offloads and omissions by study status in the choice test block. Values listed here collapse across learning strategy. For a full breakdown of values that includes learning strategy, refer to Table 4

**Table 6:** Count, expected count, and adjusted residuals for opt-outs by learning strategy

Learning strategy		Offloads	Omissions
Mnemonic	Count	141	66
	Expected count	139.92	67.08
	Adjusted residuals	0.2	−0.2
Rote	Count	199	97
	Expected count	200.08	95.92
	Adjusted residuals	−0.2	0.2

Table 6 depicts the values and outcomes of the chi-squared test of independence assessing the distribution of offloads and omissions by learning strategy in the choice test block. Values listed here collapse across learning strategy. For a full breakdown of values that includes study status, refer to Table 4

#### Decision latency

**Fig. 9:**
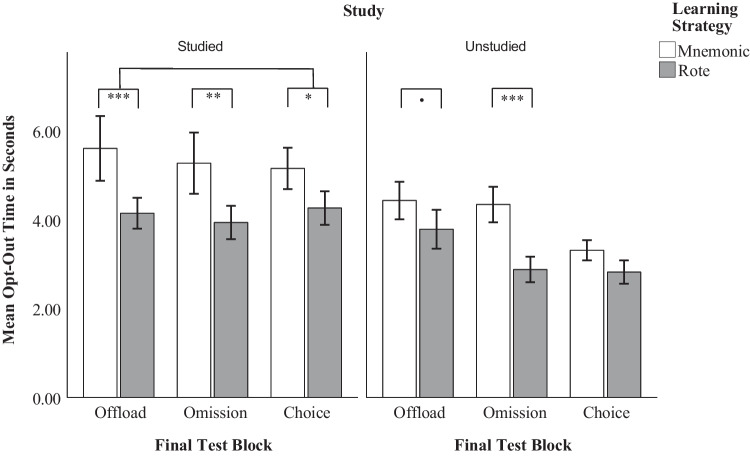
Mean response time of opt-outs in seconds by learning strategy, test block, and study status. The figure depicts the average response time of opt-out decisions in standard seconds. Offloads and omissions have been combined in the choice block, to depict the results of the latency analysis. For a full breakdown of the averaged times of offloads and omissions in the choice block, refer to Table 7. Error bars represent ± 1 SE. **.** = 0.05 > *p* < 0.10; * = *p* < 0.05; ** = *p* < 0.01; *** = *p* < 0.001

Investigating time to opt out, we used the decision time observations collapsed across matched opt-out trials in a linear mixed effects ANOVA, with learning strategy, study block, and study status predicting decision time. Data are presented in Fig. 9 and Table 7. There was a significant main effect of learning strategy on opt-out decision time, *F*(1,124.4) = 19.19, *p* < 0.001, η_p_^2^ = 0.13, with the mnemonic group thinking for longer prior to electing to opt out. Pairwise comparisons indicated that this effect was significant for studied word pairs in the offloading block (*t*(551) = 3.22, *p* = 0.001, *d* = 0.14), the omission block (*t*(566) = 2.69, *p* = 0.008, *d* = 0.11), and the choice block,* t*(522) = 2.29, *p* = 0.023, *d* = 0.10). Interestingly, this effect was also present for unstudied word pairs in the omission block (*t*(459) = 3.33, *p* < 0.001,* d* = 0.16), and was marginally significant in the offloading block (*t*(462) = 1.91,* p* = 0.057,* d* = 0.09), but not present in the choice block. There was also a significant main effect of final test block on decision time, *F*(2, 463.55) = 5.24, *p* = 0.005, η_p_^2^ = 0.02. Pairwise comparisons indicated that responses in the choice block were significantly shorter than the offloading block (*t*(468) = 3.19, *p* = 0.004, *d* = 0.15), but there were no differences between the offloading and omission blocks, and the omission and choice blocks. Lastly, there was a significant main effect of studying on response time, *F*(1, 485.67) = 71.65, *p* < 0.001, η_p_^2^ = 0.13, demonstrating that participants took longer to elect to opt out for word pairs they had studied than for unstudied word pairs in the offloading (*t*(475) = 4.32, *p* < 0.001, *d* = 0.20), omission (*t*(481) = 4.62, *p* < 0.001, *d* = 0.21), and choice blocks (*t*(471) = 6.03,* p* < 0.001,* d* = 0.28). No interactions were significant.

**Table 7:** Average time of responses by response type, learning strategy, study status, and test block

Learning strategy	Study status	Final test block	Offload	Omission	Correct	Commission
Mnemonic	Studied	Offload	5.12 (4.17)	-	2.18 (2.41)	2.75 (3.76)
		Omit	-	4.18 (3.51)	1.95 (2.92)	3.14 (4.49)
		Choice	4.46 (2.62)	3.54 (2.88)	2.14 (2.42)	2.42 (2.21)
	Unstudied	Offload	3.89 (2.62)	-	-	9.58 (10.78)
		Omit	-	3.86 (2.79)	-	0.95 (0.83)
		Choice	3.20 (1.72)	2.98 (1.73)	-	0.7 (0.28)
Rote	Studied	Offload	3.57 (2.80)	-	2.27 (3.67)	4.13 (7.03)
		Omit	-	3.24 (2.88)	1.98 (3.64)	2.08 (1.40)
		Choice	3.75 (2.67)	2.20 (1.65)	2.94 (2.98)	1.70 (2.91)
	Unstudied	Offload	3.15 (2.47)	-	-	6.88 (9.42)
		Omit	-	2.62 (1.93)	-	1.89 (1.44)
		Choice	2.51 (1.53)	2.48 (1.56)	-	5.12 (4.29)

Table 7 presents the average time of each response category, calculated at a group level. Omissions were not possible in the offloading block, and offloads were not possible in the omission block, resulting in null time values for those categories. Further, correct responses were not present for unstudied word pairs, resulting in null time values. Values are given as means, with standard errors in parentheses

Finally, in order to investigate if the learning effects were specific to the type of opt-out decision in the choice block we ran two t-tests to identify learning differences by outcome, and a regression to identify learning differences on outcome times. To perform the t-tests, we isolated responses for studied word pairs from the choice block and calculated each participants’ proportion of offloads and omissions. *P*-values for these two tests were Bonferroni corrected to account for family-wise error. There were significant differences between the mnemonic (*M* = 0.14, *SD* = 0.18) and rote groups (*M* = 0.24, *SD* = 0.26) for offloads (*t*(115.02) = −2.55, *p* = 0.024, *d* = −0.45), such that those in the rote group offloaded more than those in the mnemonic group. However, no significant differences were present between the mnemonic (*M* = 0.06, *SD* = 0.10) and rote (*M* = 0.09, *SD* = 0.21) groups for omissions. To investigate learning effects on specific outcomes, we ran an ANOVA predicting response time at a trial level by learning strategy and outcome. Learning strategy significantly affected time to opt out, with the mnemonic group searching 0.84 s longer than the rote group (*F*(1,262) = 7.26, *p* = 0.007, η_p_^2^ = 0.03). Outcome also significantly affected time to opt out, such that participants thought for 1.04 fewer seconds before omitting than they did before offloading (*F*(1,262 = 14.86, *p* < 0.001, η_p_^2^ = 0.05). Pairwise comparisons found that the learning effect was significant for omissions (*t*(262) = 2.19, *p* = 0.029, *d* = 0.10), such that the mnemonic group took 1.33 s longer to respond than the rote group. The learning effect was marginally significant for offloads (*t*(262) = 1.86, *p* = 0.063, *d* = 0.07), with the mnemonic group searching about 0.75 s longer than those in the rote group.

### Discussion

#### Offloading and omission blocks

Within the offloading-only block, we replicated the results of Experiment 1, demonstrating a significant learning strategy effect on frequency of offloading and offloading latency, supporting *H3*. However, there were no differences between the offloading and omission blocks in terms of opt-out frequency and decision latency. We therefore suggest that participants perceived both the options to offload or omit as reasonable ways to terminate a trial when they were unsure and took about the same time to decide to do so. This evidence leads us to reject *H4a*.

#### Choice block

Within the choice block, we demonstrate three main findings. Firstly, we conclude with greater confidence that participants were strategically differentially offloading, based on participants’ significant preference for offloading over omitting, and more frequent use of omissions for unstudied words than for studied. Secondly, we found that participants offloaded and omitted more frequently, and elected to do so faster, in the choice blocks than in either the offload or omit blocks. We believe this to be partially due to the availability of different types of opt-out strategies. That is, with the option to strategically offload in a way that is distinct from omitting, participants were able to seek help without terminating their search. This likely led to a diminished threshold for offloading, similar to the conclusions drawn in Ferguson et al. (2015). Instead of a binary know/don’t know outcome, participants were able to quickly answer those word pairs that they confidently knew, skip any word pairs they were confident they did not know, and focus their attention on those that they were unsure about. However, the choice block was always presented last and was the final phase of the study, so we speculate that participant fatigue may also contribute to these effects in some way. Third, we also found that despite these greater instances of offloading and omitting at shorter latencies, the learning effects on performance and latency replicated in the choice block. These findings demonstrate that participants likely searched internally for longer prior to opting out when they had learned the information using a mnemonic learning strategy, all evidence supportive of *H4b*. When broken down by type of outcome, we found that the learning strategy effect on frequency is only significant for offloads, rather than for omissions, while the learning strategy effect on latency is only suggestive for offloads, but significant for omissions.

Taken together, we find that participants will more often strategically elect to offload than omit, will offload more when they have utilized a rote strategy, and take longer about this decision when they have used a mnemonic learning strategy.

## General discussion

### Opting out

In both experiments, opt outs were more frequent in the rote group relative to the mnemonic group, demonstrating an effect of learning strategies on overall performance. Many researchers hold that increased performance from mnemonic learning is at least partially due to learned information being more distributed throughout the semantic network, with several nodes of information to draw from to recall the entire item (Bellezza, 1981; Carney & Levin, 2003; Pressley et al., 1982b), so it could be due to overall increased performance.

Regarding the decision to offload, Ferguson and colleagues (2015) presented evidence suggesting that participants were less willing to provide an answer when an offloading paradigm was present, even if they actually knew the answer. We, too, report that having access to an offloading paradigm reduces willingness to answer but specify that this is the case even when items to be recalled are familiar to the participant. We further provide novel evidence that the increased preference to offload depends on how information was learned, since opt-outs were more frequent in blocks where offloading was available, particularly the choice block. We support the growing explanation that participants, for items that they are uncertain about, will offload to check their answers when they can, but provide their best guess if their only other choice is to omit (Risko & Gilbert, 2016). In our study, participants seemed to reliably distinguish between the meaning of omissions and offloading, as they offloaded with greater frequency than they omitted, choosing to seek help rather than give up altogether. Further, in the choice block, participants were more likely to omit word pairs they had not studied than word pairs they had studied and were more likely to offload word pairs they had studied than those they did not study.

### Persisting in search

The mnemonic learning literature has reported no difference in response times for produced answers between mnemonically learned information and alternative strategies (Atkinson, 1975a; Atkinson, 1975b; Bellezza, 1981). Thus, Experiment 1’s result of no difference between learning strategies on decision latencies for correct answers and errors of commission is consistent with those findings. When considering these established findings, the observed difference between rote and mnemonic learning on errors of omission appears noteworthy: those in the mnemonic group took significantly longer prior to accessing the offloading paradigm relative to the rote group, finding that method of encoding as manipulated by learning strategy influences persisting in search behavior. The difference between learning strategies in omission response times could come from the feeling of familiarity or partial recall that accompanies the recall of the mnemonic without identifying its associated term (Adams et al., 1971; Atkinson, 1975; Bellezza, 1981; Pressley et al., 1982a). Such partial recall may prompt participants to persist in their search prior to offloading, while the rote group, with no substantial progress towards recall, would be sooner willing to offload.

It is also worth considering that Ferguson and colleagues (2015) also presented an inconsistent finding that decisions to opt out were faster when participants had an offloading paradigm relative to when they did not. They discussed that having access to the Internet adjusted the metacognitive threshold criteria for providing an answer. Interestingly, we do not replicate their timing effect between our offload and omit blocks. In fact, we find a reversal of that effect in our choice block, where participants very quickly omitted word pairs and searched longer before offloading. It is likely that both instances are true to some degree. Given that we find a learning effect on response time even for unstudied words in some blocks, it would appear that those in the mnemonic group had a greater threshold for uncertainty before opting out. Perhaps the adjustments of thresholds that participants made on the basis of their learning strategy eclipsed the adjustments of thresholds from offloading access.

Participants’ persistence in searching for items could result from several additional factors. Realistically, participants were required to give responses during the learning portion of the experiment before being able to proceed to the final test, which could have indicated to participants that providing an answer would always be required. However, other motivationally based arguments are plausible. For example, Metcalfe and colleagues (2021) suggest that curiosity can be a strong motivator towards persisting in performance tasks, while other work suggests an intrinsic value of effort expenditure that results in individual reward upon completion (Inzlicht, 2018). Thus, we suggest that participants maintained a search prior to omitting, regardless of learning strategy. The motivation behind that persistence, and especially so for each individual, is beyond the scope of the present work.

The relevance of performance in task-related decision making has been demonstrated in other research (Gilbert et al., 2020; Weis & Kunde, 2024), even some particular to offloading. Therefore, the possibility that some of the learning strategy effect on decision latency would be due to group-level differences on performance variables, such as overall frequency of offloading and study time, would be consistent with surrounding literature. However, the retention of a strong learning strategy effect on omission latency, after accounting for performance variables across two experiments, suggests that the method and quality of learning should be independently considered in the network of factors leading to a decision to offload.

The present conclusions pose further questions regarding offloading behavior during retrieval tasks for items reliably in memory. Taken together, these results suggest that strategy-based learning will influence willingness to attempt and persist in effortful search behavior rather than omitting by prolonging search times for items learned mnemonically. We suggest, accordingly, that future studies investigating offloading and memory account for the ways by which information was learned. Additionally, greater instances of omission led to decreased persistence in search, while longer study time led to greater persistence in internal search, suggesting the quality of learning plays a key role in search decisions.

## Future directions

These results indicate the importance of understanding retrieval offloading behavior in terms of the nature and degree of original storage. Given the variety of ways and degrees that participants may encode information, greater effort to differentiate offloading by retrieval task and quality of learning should allow for finer representations of the decisions that contribute to offloading. Regarding the main conclusions of this study on learning strategies, several outstanding questions remain.

We have postulated that the degree and type of encoding processes readily affect individuals’ willingness to engage in effortful internal search. In the present work, we have demonstrated that the type of encoding does influence search persistence. It would be worthwhile to continue to evaluate the relationship between the degree of encoding, willingness to offload, and metacognitive confidence. Such effects may scale well with previous research on feelings of knowing and offloading behavior (Risko et al., 2016). If individuals’ internal metacognitive cues suggest a high level of internal knowledge, they may be more likely to report a high feeling of knowing and persist longer in these searches. On the other hand, mnemonic learning is commonly reported as more effortful than rote learning; this disfluency may lead to lower confidence judgments, and as a result, greater offloading. It is also reasonable to expect that many of the additional factors that have already been established as influential in the decision to offload, such as metacognitive experiences (Hu et al., 2019; Risko et al., 2016) or beliefs about the offloading agent (Weis & Weise, 2022) could nuance the observed relationship. Parsing the relative contributions of all these factors to offloading behavior should provide useful information. Additionally, future studies could include evaluating the learning effect on offloading over longer retention intervals. For example, following forgetting within the first 24 h, participants may generally feel less confident in their retrieval ability, isolating the learning effect more significantly.

The effect that performance influenced offloading latency is of current relevance in offloading literature and can be viewed from several different perspectives. Recently, Weis and Kunde (2024) demonstrated that performance differences motivated most offloading decisions, such that participants chose evenly between internal and external strategies when performance was held constant. This finding provided tangible evidence that, with generally high performance in an existing strategy, individuals are likely to persevere in using that strategy. Additionally, literature regarding sets of decision-making guidelines known as stopping rules may be of use when contextualizing the present findings. For example, that research has demonstrated that as participants experience more retrieval failures, they reduce the threshold for time spent in internal search before terminating the internal search (Dougherty et al., 2014; Hussey et al., 2014). Behavioral findings such as this may be able to inform settings where searches are extended to external stores, rather than simply terminated. Lastly, Ackerman (2014) has demonstrated a form of metacognitive stopping rules, in an effort to explain differing relationships between time invested in a task and confidence judgments. They suggested that participants invest time and effort into completing a task to a certain goal level, and the continuation of investment beyond expectations results in compromising characteristics of the goal state level. Ackerman (2014) additionally found that intermediate levels of metacognitive confidence and perceived progress on the task contribute to the degree of compromise participants express. All three of these perspectives elucidate the ways that participants are more willing to abandon their efforts as performance decreases and suggest that this decrease is incremental over prolonged time on task. Such explanations could hold heavy influence in the ways that performance affects persistence in internal searches and should be an area of future direction.

Further, understanding the characteristics of partially learned or learned but forgotten information should allow us to test for nuances in searching and offloading behavior. For example, previous experience with the information which the individuals are searching for may influence their ability to restrict searches for complex information (e.g., using JSTOR databases) or their desire to engage in checking behavior. As future research parses such conditional learning, we may be able to identify categories or particularities within offloading behavior.

## Limitations

Using the experimenter-provided simulated Google Translate screenshot to maintain consistent veracity in offloading behavior may also have influenced the choices participants made. Marsh and Rajaram (2019) conclude that a major factor of the Internet is a requirement to actively search for an item through search engines. Here, the activity of offloading minimized that search to one step. By minimizing the required effort of offloading, it is possible that opt-out responses were inflated, but we find this suggestion unlikely, given the generally high performance of both learning strategies in terms of correct answers. This could be studied by manipulating the difficulty of the search task (e.g., adding more steps; restricting search terms) to examine the effect on offloading behavior.

Additionally, general mnemonic research suggests that recall takes part in two steps: first the recall of the mnemonic, and second the decoding of its associated target term (Pressley et al., 1982a). In this study, we suggest that partial recall of the response term after successful recall of the mnemonic is possible and could lead to longer internal search times prior to offloading. That is, the participant has some glimmer of hope to retrieve the correct response, and so persists. However, we did not measure recall of the mnemonic; rather, we were interested principally in the time to offload. Further research could examine the time differences between start of search, recall of the mnemonic, and eventual offloads for explicitly partial mnemonic retrieval.

We also acknowledge that there are many ways to form mnemonic associations, including visual imagery associations (Bellezza, 1981; Cohen, 2008; Cook, 2023; McCarty, 2008; Paivio, 1969, Thomas, et al., 2023). The instructions and task requirements in this study were exclusively verbal, and we did not significantly attempt to control for or identify types of other mnemonic strategies used. It is possible that efforts to parse the relationship between types of mnemonic learning strategies and offloading decisions may yield fruitful nuances.

## Final statement

In the present work, we find that across two paired associate recall tasks, participants volunteer more responses and persist longer before opting out after using a mnemonic rather than rote learning strategy. The data from Experiment 1 also suggest that the learning strategy effect on decision latency is independent of additional online performance effects, like frequency of opting out and study time. Data from Experiment 2 indicate that the learning strategy effect is specific to the frequency of strategic offloading decisions, rather than generic decisions to opt out. This work adds to our understanding of the conditions and ways people persist in effortful search in the presence of an offloading paradigm.

## Data Availability

Materials and datasets generated and/or analyzed during the current study are available from the corresponding author upon reasonable request. Neither experiment was preregistered.

## Appendix A

**Table:**

Full item list, Experiment 1		
potato – aardap	hire – aaname	ask – vragen
highway – snelwe	happen – gebeur	lose – verlie
fridge – koelka	accountant – boekho	marry – trouwe
shop – winkel	autumn – herfst	feel – voelen
drive – rijden	canal – gracht	holiday – vakant
write – schrij	relationship – verwan	sunglasses – zonneb
run – hardlo	vegetable – groent	close – sluite
decide – beslis	look – kijken	travel – reizen
Full item list, Experiment 2		
List A	List B	List C
Studied:	Studied:	Studied:
belt – riem	think – denk	stool – kruk
mustache – snor	believe – gelo	sunglasses – zonn
close – slui	travel – reiz	highway – snel
ask – vrag	robe – jurk	keep – houd
accountant – boek	look – kijk	holiday – vaka
pants – broe	lose – verl	autumn – herf
evening – avon	canal – grac	feel – voel
job – baan	borrow – lene	fridge – koel
Unstudied:	Unstudied:	Unstudied:
bike – fiet	happen – gebe	art – kuns
drive – rijd	garden – tuin	decide – besl

## Appendix B

### Supplemental Results

Table 8

**Table 8:** Response time by learning strategy and response type in logged seconds

Response time (Logged)				
Group	Correct	Opt-Out	Commission	Total
Mnemonic	0.91 (0.05)	2.53 (0.08)	0.92 (0.07)	1.45 (0.04)
Rote	0.91 (0.04)	2.11 (0.07)	0.85 (0.07)	1.29 (0.04)
Total	0.91 (0.03)	2.32 (0.05)	0.88 (0.05)	-

## Footnotes

1 JOL data missed about 40% of responses due to participant disengagement. This disengagement did not appear to carry over to performance during the remainder of the learning and testing trials. For this reason, JOL data were excluded from the analysis to maintain power for the main hypotheses.

2 Given that the study and its outlier procedures were not preregistered, we did perform the analysis with all outliers included. When performed with the transformations described above, all effects replicated with the exception of the interaction between learning strategy and response type, which approached significance (*F*(2,120) = 2.25, *p* =.10, η_p_^2^ =.02). However, when investigating the pairwise comparisons, the group difference for the omitted responses was significant (*F*(1,121) = 10.88, *p* =.001, η_p_^2^ = 0.08). We proceed with the presented analysis as it boosts power on the marginally significant effect and aids in reducing negative skew on affected variables, which extreme outliers were contributing towards.

## Code availability

Stimulus presentation was through Qualtrics software. Custom code for data preparation is available from the corresponding author upon reasonable request.
